# Bone resorption by osteoclasts involves fine tuning of RHOA activity by its microtubule-associated exchange factor GEF-H1

**DOI:** 10.3389/fphys.2024.1342024

**Published:** 2024-01-19

**Authors:** Anne Morel, Christophe Douat, Anne Blangy, Virginie Vives

**Affiliations:** CRBM (Montpellier cell Biology Research Center), Univ Montpellier, CNRS (National Center for Scientific Research), Montpellier, France

**Keywords:** bone, osteoclast, resorption, sealing zone, microtubules, cytoskeleton, GEF-H1

## Abstract

Bone health is controlled by the balance between bone formation by osteoblasts and degradation by osteoclasts. A disequilibrium in favor of bone resorption leads to osteolytic diseases characterized by decreased bone density. Osteoclastic resorption is dependent on the assembly of an adhesion structure: the actin ring, also called podosome belt or sealing zone, which is composed of a unique patterning of podosomes stabilized by microtubules. A better understanding of the molecular mechanisms regulating the crosstalk between actin cytoskeleton and microtubules network is key to find new treatments to inhibit bone resorption. Evidence points to the importance of the fine tuning of the activity of the small GTPase RHOA for the formation and maintenance of the actin ring, but the underlying mechanism is not known. We report here that actin ring disorganization upon microtubule depolymerization is mediated by the activation of the RHOA-ROCK signaling pathway. We next show the involvement of GEF-H1, one of RHOA guanine exchange factor highly expressed in osteoclasts, which has the particularity of being negatively regulated by sequestration on microtubules. Using a CRISPR/Cas9-mediated GEF-H1 knock-down osteoclast model, we demonstrate that RHOA activation upon microtubule depolymerization is mediated by GEF-H1 release. Interestingly, although lower levels of GEF-H1 did not impact sealing zone formation in the presence of an intact microtubule network, sealing zone was smaller leading to impaired resorption. Altogether, these results suggest that a fine tuning of GEF-H1 through its association with microtubules, and consequently of RHOA activity, is essential for osteoclast sealing zone stability and resorption function.

## Introduction

Bone is a highly dynamic tissue whose permanent remodeling is mediated by the balanced actions of bone-forming osteoblasts and bone-resorbing osteoclasts. Increased bone resorption by osteoclasts leads to pathological decreased bone density characteristic of osteolytic diseases such as post-menopausal osteoporosis, rheumatoid arthritis or bone metastasis (Yokota, 2023). Osteoclasts are multinucleated cells derived from the fusion of mononuclear hematopoietic progenitors of the myeloid lineage in a multi-step process termed osteoclastogenesis (Sun et al., 2021). Their unique ability to degrade bone tissue relies on superficial mineral phase dissolution by H^+^ and Cl^−^ secretion allowing collagen-rich underlying organic phase digestion by subsequently released lysosomal proteases (Everts et al., 2022). For optimal bone resorption efficiency, osteoclasts form a specialized cell-matrix adhesion structure, called sealing zone or actin ring that creates a confined acidic microenvironment over the area that needs to be resorbed. This dynamic actin-rich structure is composed of densely packed podosomes locally connected by acto-myosin filaments and surrounded by adhesion complexes (Luxenburg et al., 2007; Portes et al., 2022). It is intimately connected to a network of microtubules (Akisaka et al., 2011), and microtubule depolymerization in osteoclasts disrupts the sealing zone and inhibits their resorption activity (Destaing et al., 2003; Okumura et al., 2006). In osteoclasts, podosomes interaction with microtubules was shown to be regulated directly by adaptors, such as unconventional myosin X (McMichael et al., 2014) and dynamin 2 GTPase (Batsir et al., 2017) as well as indirectly by Pyk2-RHOA-mDia2-HDAC6 axis. During osteoclast maturation, the activity of RHOA has to be maintained at low level by Pyk2 to allow the sealing zone stabilization and bone resorption (Gil-Henn et al., 2007). Indeed, constitutively active RHOA promotes significant microtubules deacetylation resulting in podosomes disorganization. More, RHOA inhibition by TAT-C3 was shown to prevent the nocodazole-induced adhesion structure destabilization (Destaing et al., 2005). RHOA is therefore a key player in the crosstalk between actin cytoskeleton and microtubules network, but the underlying mechanisms linking RHOA activation to microtubule destabilization are still unclear.

RHOA, a member of the Rho family of small GTPases, is activated by numerous Guanine nucleotide Exchange Factors (GEF) that catalyze the exchange of GDP for GTP (Haga et al., 2016). Among them, GEF-H1, a member of the Dbl family, also known as ARHGEF2 and Lfc, stands out for its regulation by microtubules (Joo et al., 2021). It was shown in various cell types that GEF-H1 is sequestered on polymerized microtubules by dynein motor light chain protein Tctex-1 (DYNLT1) where it is held in an inactive state through Ser^885 (mouse)/886 (human)^ phosphorylation by various kinases such as PAK1 (Zenke et al., 2004), Par1 (Yamahashi et al., 2011) or PKA (Meiri et al., 2012). This phosphorylation generates a docking site for 14-3-3 which holds GEF-H1 in a catalytically inactive configuration (Meiri et al., 2009). GEF-H1 sequestration on microtubules prevents its physical interaction with RHOA and subsequent activation of the GTPase. GEF-H1 release from the microtubule array can be triggered by either microtubule depolymerization by nocodazole (Krende and, Zenke, 2002; Ito et al., 2017), microtubule destabilization (Nagae and Meng, 2013; Law et al., 2023) or by its dissociation from DYNLT1 (Kakiashvili et al., 2009; Kakiashvili et al., 2011; Meiri et al., 2014; Sandí et al., 2017). More, reduced expression of GEF-H1 impacted the assembly rate of Src-induced podosomes in NIH 3T3 invadopodia (Shiba et al., 2011). Our previous reports revealed that GEF-H1 mRNA and protein are expressed in osteoclasts (Brazier et al., 2006; Guérit et al., 2020), but its function was unexplored in this cell type so far.

In the present study, we investigated the hypothesis that GEF-H1 was an important regulator of RHOA activity in mature osteoclasts as part of the crosstalk between actin cytoskeleton and microtubules. On one hand, we report that GEF-H1 release upon microtubules depolymerization activated RHOA and its effector ROCK, leading to podosome disorganization in osteoclast adhesion structure. On the other hand, we show that GEF-H1 is essential for osteoclast resorption function. Thus, fine tuning of GEF-H1 activity by microtubules is key to control the activity of RHOA and actin organization in osteoclasts.

## Materials and methods

### Chemicals

Nocodazole (#M1404) was purchased from Sigma Aldrich and Y27632 from MCE (#HY-10071).

### Ethics statement

Mice sacrifice and bone marrow harvest were performed in compliance with local animal welfare laws, guidelines and policies, according to the rules of the regional ethical committee.

### Bone marrow macrophages isolation and differentiation in osteoclasts

Primary osteoclasts were obtained from 6- to 8-week-old C57BL/6J mice as previously described (Morel et al., 2018). Briefly, bone marrow macrophages (BMM) were obtained from long bones by growing non-adherent cells for 48 h in *α*MEM containing 10% heat-inactivated foetal calf serum (Biowest), 2 mM glutamine, 100 U/mL penicillin–streptomycin and 30 ng/ml M-CSF (Miltenyi, #130-101-703). Osteoclasts were then differentiated by culturing BMM in the same medium supplemented with 50 ng/mL RANKL (Miltenyi, #130-094-076) for 4–6 days.

### Generation of GEF-H1 knock-out RAW 264.7 cells

A guide RNA (gRNA) targeting exon 13 of mouse GEF-H1 gene *Arhgef2* (5′-AGG​ATA​AGG​CGT​ATC​TCC​GGA​GG-3′) was designed and cloned in lentiCRISPRv2 vector, which also provides the expression of the *Streptococcus pyogenes* Cas9 and puromycin resistance, a gift from Feng Zhang (Addgene plasmid # 52961; http://n2t.net/addgene:52961; RRID:Addgene_52961) (Sanjana and Shalem, 2014). Empty and GEF-H1 gRNA-containing lentiCRISPRv2 were used to produce lentiviruses and generate respectively control (CTL) and GEF-H1 Knock-Out (KO) RAW264.7 cells. Briefly, growing RAW264.7 cells, a gift from Kevin P McHugh (Gainesville, FL, United States), were infected with lentiviral particles and selected with 3 mg/mL puromycin 48 h later. Puromycin resistant CTL and GEF-H1 KO RAW264.7 clones were individually picked and expanded. GEF-H1 expression was monitored by immunoblot analysis to select clones with reduced GEF-H1 expression.

### Raw264.7 cell culture and differentiation in osteoclast

Wild-type and CRISPR/Cas9 modified RAW264.7 cells were grown in DMEM containing 10% heat-inactivated foetal calf serum (Eurobio) with 2 mM glutamine and 100 U/mL penicillin–streptomycin. For osteoclast differentiation, they were seeded at 5 × 10^4^ cells/well (6-well plate) or 8 × 10^3^ cells/well (24-well plate) in *α*MEM containing 10% heat-inactivated fetal calf serum (Biowest), 2 mM glutamine, 100 U/mL penicillin–streptomycin and 50 ng/mL of RANKL (Miltenyi, #130-094-076) for 3–4 days. Since pure GEF-H1 KO clones did not differentiate, 75% GEF-H1 KO clones had to be mixed with 25% CTL RAW264.7 cells to fuse and form GEF-H1 knock-down (KD) osteoclasts. Osteoclasts were imaged with an EVOS FL microscope equipped with a Sony ICX445 CCD camera.

### Apatite collagen complex (ACC)-coated substrate preparation and osteoclast seeding

The ACC preparation protocol was simplified from (Saltel et al., 2004). Briefly, 6-well plates or 13 mm diameter glass coverslips were coated with 50 μg/mL calf skin type I collagen (Sigma, #C9791) in 20 mM acetic acid, incubated for 1 h at 37°C and dried overnight. Then supports were successively incubated in (1) 200 mM Tris-buffered saline (TBS) pH 9 containing 0.13 mg/mL egg yolk phosvitin (Sigma, #P1253), 0.13 mg/mL alkaline phosphatase (Sigma, #P764) and 1 mg/mL dimethyl suberimidate dihydrochloride (Sigma, #179523) as a cross-linking reagent during 24 h at 37°C, (2) 6 mM calcium β-glycerophosphate (Sigma, #G6626) for 48 h at 37°C and (3) washed with 200 mM Tris pH 9. The last 3 steps were repeated 3–4 times depending on the amount of precipitated calcium phosphate. Next, the supports were rinsed with distilled water and air dried. Osteoclasts at day 3 of differentiation were detached with Accutase (Sigma, #A6964), scrapped, seeded and grown for 2 more days onto ACC.

### Microtubule sedimentation assay

The assay was performed as previously described (Ito et al., 2017). Cells were lysed with a microtubule stabilizing buffer (100 mM PIPES pH 6.8, 1 mM MgSO4, 1 mM EDTA, 2 M glycerol, 0.1% (w/v) Triton X-100 and protease inhibitor cocktail) for 20 min at 37°C and centrifuged at room temperature for 5 min at 16,000 g. The supernatant was collected as the soluble fraction. The pellet was lysed with a whole cell lysis buffer (10 mM Tris-HCl pH 7.5, 2 mM EDTA, 1% SDS and protease inhibitor cocktail), boiled for 15 min, further centrifuged at room temperature for 5 min at 20,000 g and used as the insoluble fraction.

### RHOA activity assay

The RHOA-binding domain of Rhotekin (RBD) pulldown assay was used to detect cellular GTP bound RHOA. In brief, osteoclasts were washed with cold TBS and lysed in a cold buffer containing 50 mM Tris-HCl pH 7.5, 1% triton, 500 mM NaCl, 10 mM MgCl_2_, 1 mM DTT and protease inhibitor cocktail. After centrifugation at 13,000 g for 2 min at 4°C, part of the supernatant was stored for total RHOA determination. The remaining supernatant was incubated with 66 µg of GST-RBD-coupled gluthatione sepharose beads (cytoskeleton, #RT02) for 45 min at 4°C. The beads were washed 3 times with a cold buffer containing 50 mM Tris-HCl pH 7.5, 0.5% triton, 150 mM NaCl, 10 mM MgCl_2_, 1 mM DTT and protease inhibitor cocktail. Total and active GTP-bound RHOA were detected by Western blotting.

### Western blot

Whole cell extracts were prepared in Laemmli sample buffer, resolved on SDS-PAGE and electrotransferred on PVDF membranes (Millipore, #IPFL00010). Immunoblotting was performed using the following primary antibodies: rabbit anti-GEF-H1 (Abcam, #ab155785, 1/500), mouse anti-alpha tubulin (Sigma, #T6074, 1/1000), rabbit anti-GAPDH (Cell Signaling, #2118, 1/2000), rabbit anti-histone H3 (Abcam, #1791, 1/5000) and mouse anti-RHOA (Santa Cruz, #sc-418, 1/500). Signals were revealed with Dylight 680 or 800 conjugated secondary antibodies (Invitrogen) using the Odyssey Infrared Imaging system and then quantified with Image Studio software (LI-COR).

### Immunofluorescence

Osteoclasts on 13-mm diameter glass or ACC-coated coverslips were either fixed for 20 min in 3.2% paraformaldehyde in PHEM (60 mM Pipes, 25 mM Hepes, 10 mM EGTA, 4 mM MgSO4, pH 6.9), permeabilized with 0.1% Triton X100 for 1 min and blocked with 1% BSA in PBS for 15 min or permeabilized and blocked with PBS - 2% BSA - 0.2% triton X100 for 1 h (GEF-H1 staining). Then osteoclasts were incubated for 1 h with primary antibodies: rabbit anti-GEF-H1 (Abcam, #ab155785, 1/200), mouse anti-alpha tubulin (Sigma, #T-5168, 1/2000) and/or Alexa 564-phalloidin (Life Technologies, #A22283, 1/1000). Signal was revealed with the adapted Alexa Fluor 488-, 546 or 647-conjugated secondary antibodies (Life Technologies, 1/1000). Preparations were mounted in Citifluor mounting medium (Biovalley) and imaged with Leica SP5-SMD confocal microscope using 40X HCX Plan Apo CS oil 1.3NA or 63X HCX Plan Apo CS oil 1.4NA objectives. Co-localization was shown using ImageJ pluggin DiAna (Gilles et al., 2017).

### Osteoclast activity assay

Mineral dissolution activity of osteoclasts was measured as described (Morel et al., 2018). Briefly, at day 3 of differentiation, osteoclasts were rinsed once in PBS, detached with Accutase for 5–10 min at 37°C, scrapped, seeded and grown for 2 more days onto inorganic crystalline calcium phosphate (CaP)-coated multiwells (Osteo Assay Surface, Corning). In each experiment, four wells were stained for Tartrate Resistant Acid Phosphatase (TRAP) activity to count osteoclasts and four wells with Von Kossa stain to measure CaP dissolution. They were imaged with an EVOS FL microscope equipped with a Sony ICX445 CCD camera and quantification of osteoclasts and resorbed areas were done with ImageJ 1.53w software. Osteoclast specific activity was expressed as the average area resorbed in the wells stained with von Kossa normalized by the average area of osteoclasts in the wells stained with TRAP.

### Statistical analysis

All analyses were performed using GraphPad Prism 5.01 (GraphPad Software, Inc., La Jolla, CA). All data are presented as the mean ±sem; p values <0.05 were considered statistically significant.

## Results

### Microtubules modulate the RHOA-ROCK pathway for the maintenance of podosome organization in osteoclasts

**FIGURE 1:**
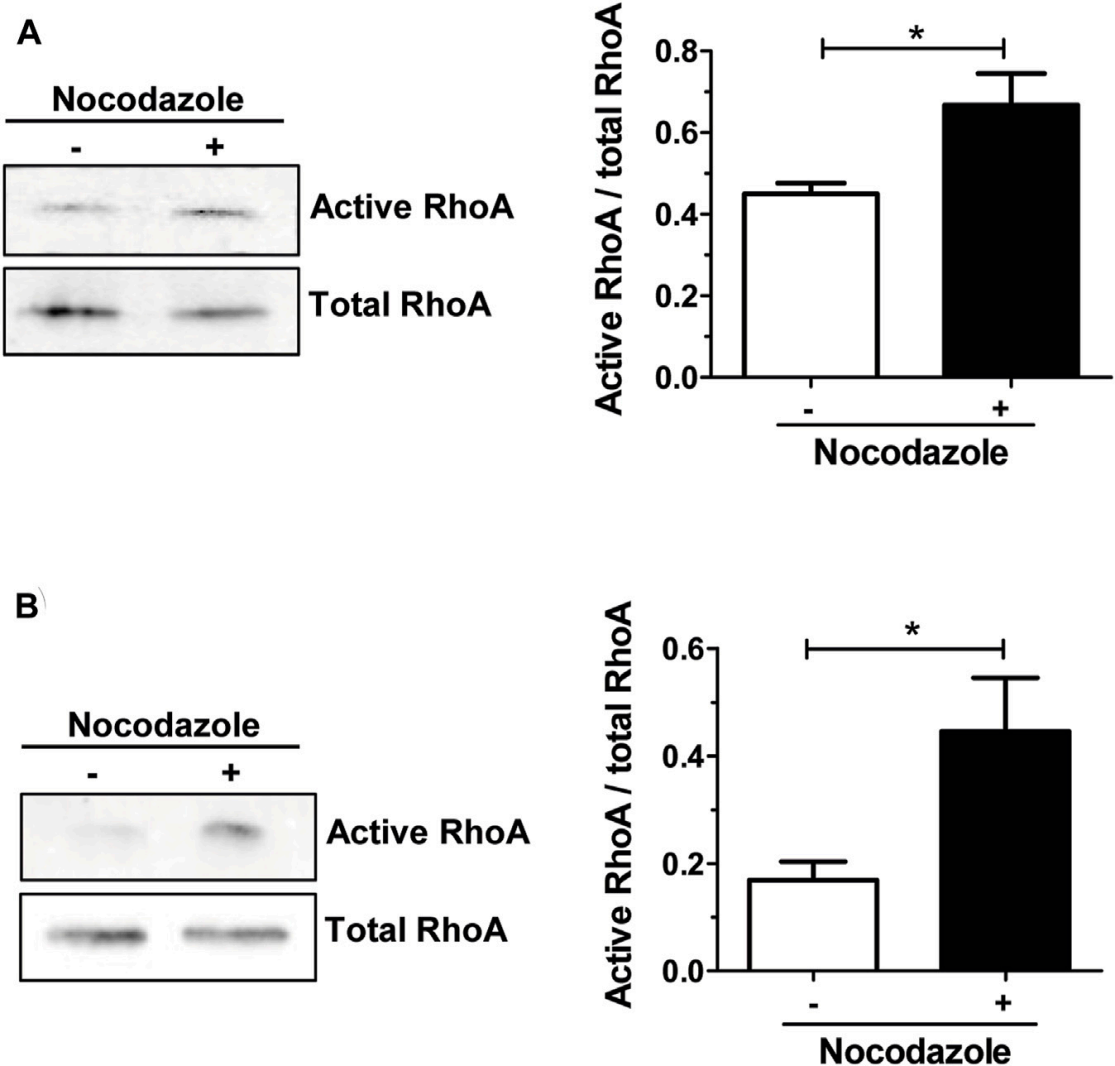
Nocodazole treatment activates RHOA in osteoclasts. Representative Western blot showing active and total RHOA from wild-type primary osteoclasts sitting either on plastic **(A)** or on mineral matrix **(B)** and treated (+) or not (−) with 10 µM nocodazole for 1 h (left). Normalized active RHOA mean level ±s.e.m. from five **(A)** or four **(B)** independent experiments was represented on a bar graph (right). *, *p <* 0.05 as determined by two-tailed Mann-Whitney test.

In osteoclasts, stabilization of the unique podosomes organization by microtubules is instrumental for bone resorption function (Blangy et al., 2020). Microtubule depolymerization with nocodazole causes the rapid collapse of the adhesion structure (Destaing et al., 2003), but the underlying mechanisms linking actin cytoskeleton and microtubules in osteoclasts remain largely unknown. It was shown that a tight control of RHOA activity is required for the unique patterning of podosomes in osteoclast (Touaitahuata et al., 2014) and that RHOA inhibition by TAT-C3 prevented the destabilization of the podosome belt by nocodazole (Destaing et al., 2005). Since the depolymerization of microtubules increased RHOA activity in various cell types (Kee et al., 2002) (Chang et al., 2006; Chang et al., 2008; Ito et al., 2017; Takesono et al., 2010), we tested whether it was also the case in osteoclasts. To do so, we treated mouse bone marrow macrophages (BMM)-derived osteoclasts with nocodazole and performed RHOA pull-down assay to monitor the activity of the GTPase. When osteoclasts were seeded on plastic, we observed a significant 1.5-fold increase in RHOA activity after 1-hour nocodazole treatment, as compared to the control (Figure 1A). Moreover, when osteoclasts were seeded on mineralized apatite collage complex (ACC), RHOA basal activity was higher and increased by 3-fold after the same nocodazole treatment (Figure 1B). Thus, the depolymerization of microtubules leads to the activation of RHOA in osteoclasts.

**FIGURE 2:**
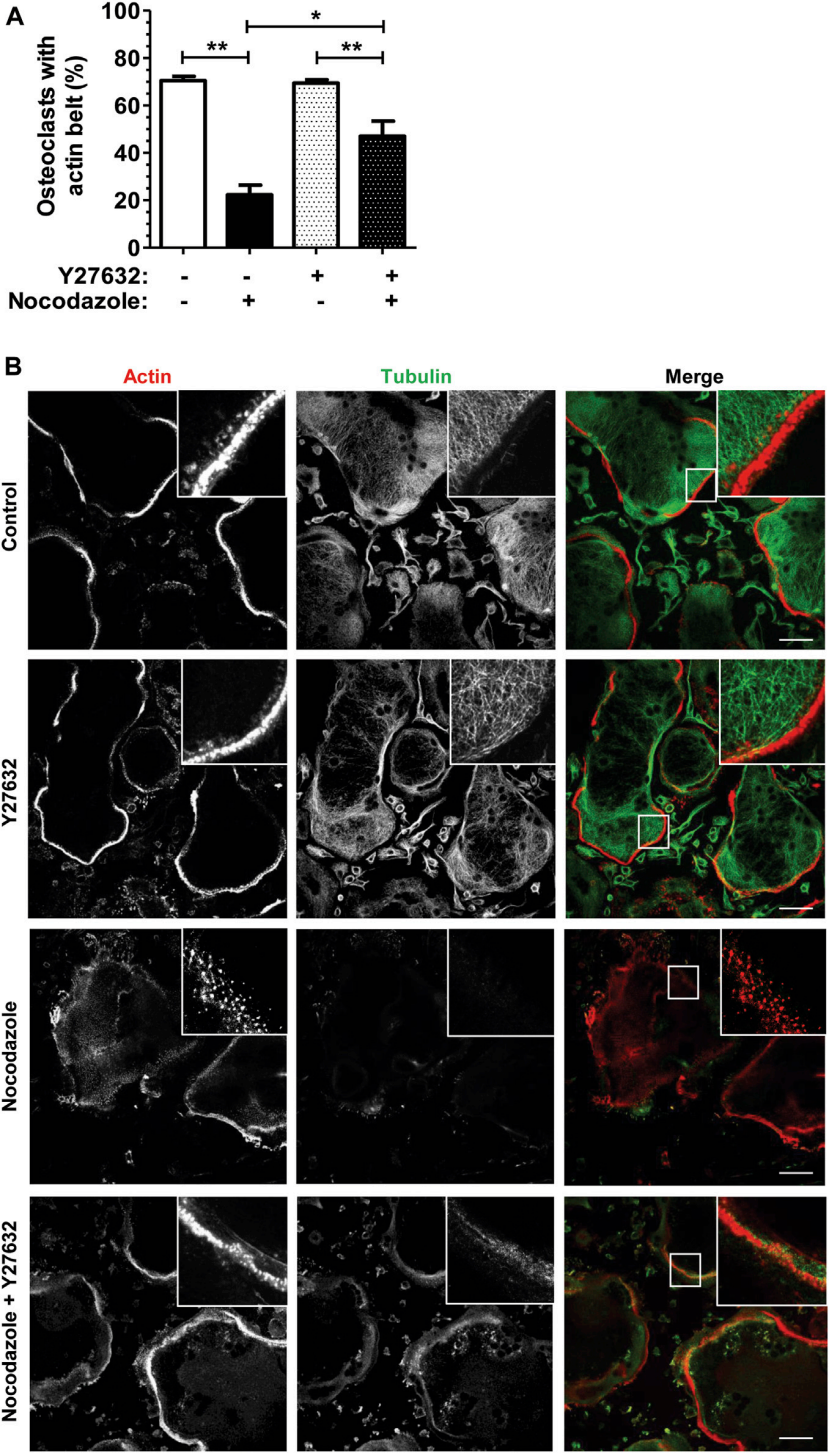
ROCK kinase inhibition partially recues nocodazole-dependant podosome belt loss. **(A)** Bar graph showing mean percent ±s.e.m. of wild-type primary osteoclast with podosome belt treated (+) or not (−) with 10 µM nocodazole and 30 µM Y27632 for 1 h (*n* = 6 independent experiments). *, *p <* 0.05; **, *p <* 0.01 as determined by two-tailed Mann-Whitney test. **(B)** Confocal images showing representative podosome belts in wild-type primary osteoclasts sitting on glass depending on the treatment. Osteoclasts were labelled for actin (red) and tubulin (green). Insets in images show high magnifications of boxes areas. Scale bars: 50 µm.

To confirm that the nocodazole-induced disorganization of osteoclast actin cytoskeleton was mediated by RHOA activation, we used Y-27632, an inhibitor of the ROCK kinase, a major effector of RHOA downstream signaling. Osteoclasts plated on glass do not form a *bona fide* sealing zone but a thinner peripheral structure called the podosome belt, which is characterized by a lower density of podosomes (Luxenburg et al., 2007). Treatment of osteoclasts with Y-27632 alone did not affect podosome organization (Figures 2A, B). More interestingly, Y-27632 was able to prevent the nocodazole-induced disorganization of the podosome belt (Figures 2A, B). Thus, RHOA activation upon microtubule depolymerization in osteoclasts contributes to the disorganization of the podosomes, via the activation of ROCK kinase.

Overall, these results suggest that microtubules have a key role in osteoclasts for the maintenance of actin cytoskeleton organization thought the modulation of the activity of the GTPase RHOA.

### GEF-H1 is associated with osteoclast microtubules

We reported previously SILAC proteomic and RNAseq transcriptomic data showing that the mRNA of the RHOA exchange factor GEF-H1 was expressed at high levels in osteoclasts, with comparable levels of mRNA and proteins in BMM and osteoclasts (Guérit et al., 2020). We also reported proteomic data indicating that GEF-H1 is present in microtubule-associated protein enriched fraction of osteoclasts derived from RAW264.7 cells (Maurin et al., 2021). In various epithelial cancer cell lines, it was shown that nocodazole treatment induced the activation of RHOA due to the release GEF-H1 from the microtubules to which it is basally anchored and held in an inactive state (Meiri et al., 2012). This suggests that GEF-H1 could be associated with microtubules in BMM-derived osteoclasts and mediate the activation of RHOA upon microtubule depolymerization.

**FIGURE 3:**
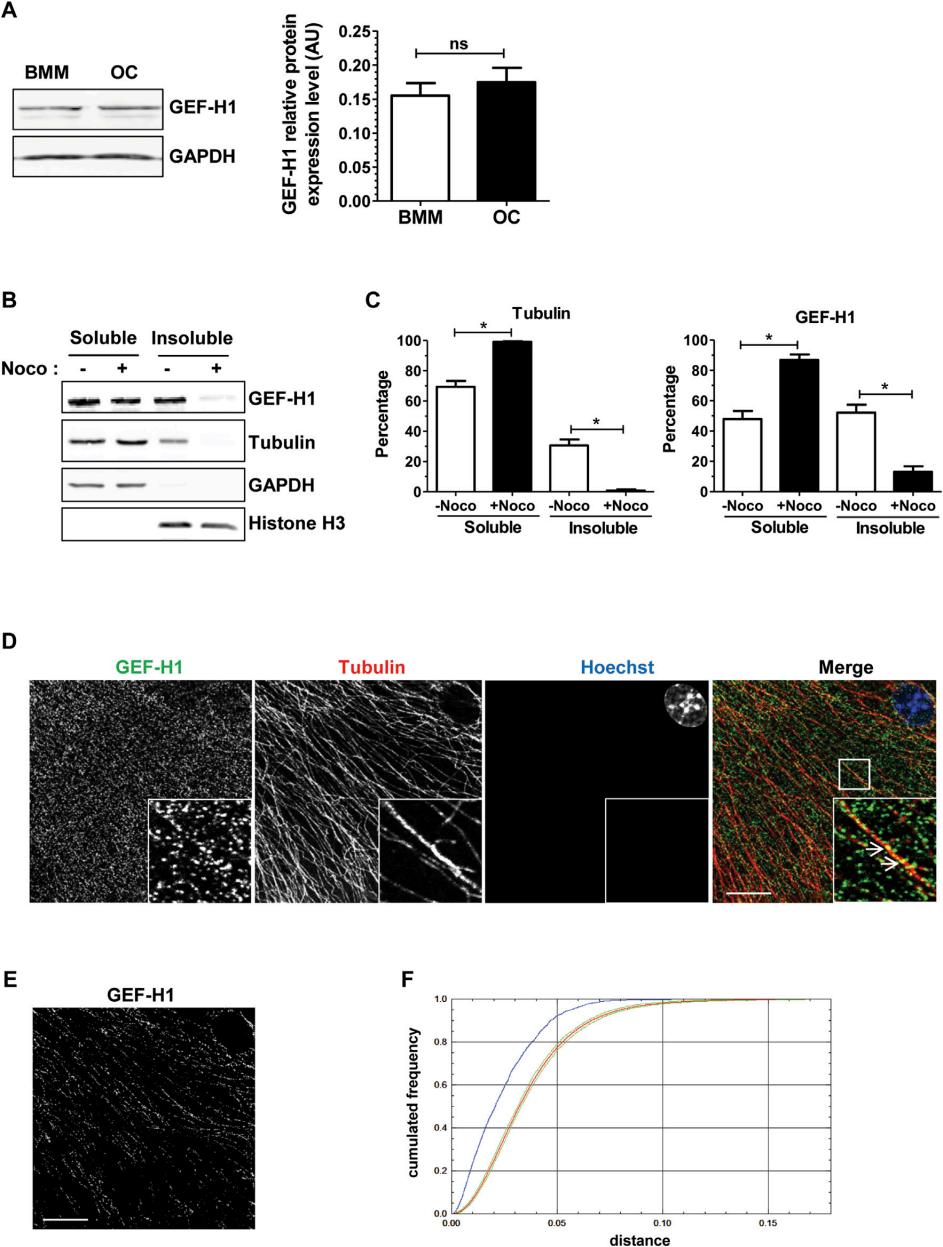
GEF-H1 is partly localized on osteoclast microtubules. **(A)** Representative western blots (left) showing GEF-H1 and GAPDH expression levels in mouse bone marrow macrophages differentiated (OC) or not (BMM) in osteoclasts. Bar graph (right) showing GEF-H1/GAPDH mean protein level ±s.e.m. ns, not significant as determined by two-tailed Mann-Whitney test. **(B)** Representative western blots showing GEF-H1 and tubulin presence in soluble or insoluble fraction from wild-type primary osteoclasts treated (+) or not (−) with 10 µM nocodazole for 1 h. GAPDH and histone H3 are respectively controls of soluble and insoluble fraction purity. **(C)** Bar graphs showing tubulin (left) and GEF-H1 (right) protein mean percentage ±s.e.m. in soluble and insoluble fractions treated (+Noco) or not (−Noco) with 10 µM nocodazole for 1 h (*n* = 3 independent experiments). *, *p <* 0.05 as determined by two-tailed Mann-Whitney test. **(D)** Confocal images of a single plane showing GEF-H1 (green) co-localization with tubulin (red) in wild-type osteoclasts labelled with Hoechst (blue). Insets in images show high magnifications of boxes area. White arrows point toward GEF-H1 association with microtubules. Scale bar: 10 µm. **(E)** Image showing GEF-H1 staining from **(D)** that co-localizes with microtubules. Scale bar: 10 µm. **(F)** Graphic representing the cumulative distribution of the minimum distances between GEF-H1 staining and microtubules from **(D)**. The red curve shows the mean cumulative distribution of GEF-H1 staining redistributed in a uniform manner in 100 images flanked by 95% intervals of the results (green curves). The blue curve shows the distribution of distances non-randomized GEF-H1 staining and microtubules. Since it is localized outside the confidence interval of the distance analysis done after randomization, GEF-H1 co-localization to microtubules is considered statistically significant. The same result was obtained with 3 other images.

We confirmed by Western blot on total cell extracts that GEF-H1 was expressed at comparable levels in BMM and osteoclasts (Figure 3A). We then examined whether GEF-H1 was indeed associated with osteoclast microtubules. First, we performed a microtubule sedimentation assays on BMM-derived osteoclasts in the absence or presence of nocodazole. In control conditions, GEF-H1 was predominantly in the microtubule-containing insoluble fraction of osteoclasts; in contrast, the protein mostly shifted in the soluble fraction, when the fractionation was performed in the presence of nocodazole, similar to the behavior of β-tubulin used as a marker of microtubule depolymerization (Figures 3B, C). These results are consistent with the hypothesis that GEF-H1 associates with osteoclast microtubules, and thus could be released upon nocodazole treatment. By confocal fluorescent microscopy, we confirmed that GEF-H1 colocalizes with microtubules (Figures 3D–F).

These data show that the RHOA exchange factor GEF-H1 associates with osteoclast microtubules; they also suggest that the activation of RHOA we observed upon osteoclast microtubule depolymerization could be mediated by GEF-H1 release from its inhibitory association with microtubules.

### GEF-H1 is involved in RHOA activation and actin cytoskeleton disorganization in response to microtubule depolymerization

To be able to examine the role of GEF-H1 in osteoclasts and its regulatory action on the activity of the GTPase RHOA, we generated a GEF-H1 knock-out model by CRISPR/Cas9-mediated genome editing of mouse monocytic RAW264.7 cell line, using a guide RNA to target *Arhgef2* exon 12. As a control, RAW264.7 cells were treated the same with an empty guide RNA vector. We selected two clones based on GEF-H1 expression level: on one hand, clone KO#5 presented a one-nucleotide frameshift insertion or deletion in each allele, resulting in no expression of GEF-H1; on the other hand, clone KO#26 presented a 169 out of frame deletion on one allele, resulting in reduced levels of GEF-H1, as compared to CTL cells (Supplementary Figure S1). Incubation of the cells with RANKL revealed that neither clone #5 nor clone #26 was able to form multinucleated cells, in contrast with CTL RAW264.7 cells. Mixing 75% of KO#5 or KO#26 with 25% CTL cells rescued multinucleated cell formation (Supplementary Figures S2A, B), leading to normal-sized osteoclasts as compared to CTL (Supplementary Figure S2C). These data show that minimal levels of GEF-H1 are required for the differentiation of osteoclasts. From now on, knock-down osteoclasts expressing lower levels of GEF-H1 compared to CTL, KD#5 and KD#26, were studied after differentiation of a mixture of 75% KO and 25% CTL RAW264.7 cells.

**FIGURE 4:**
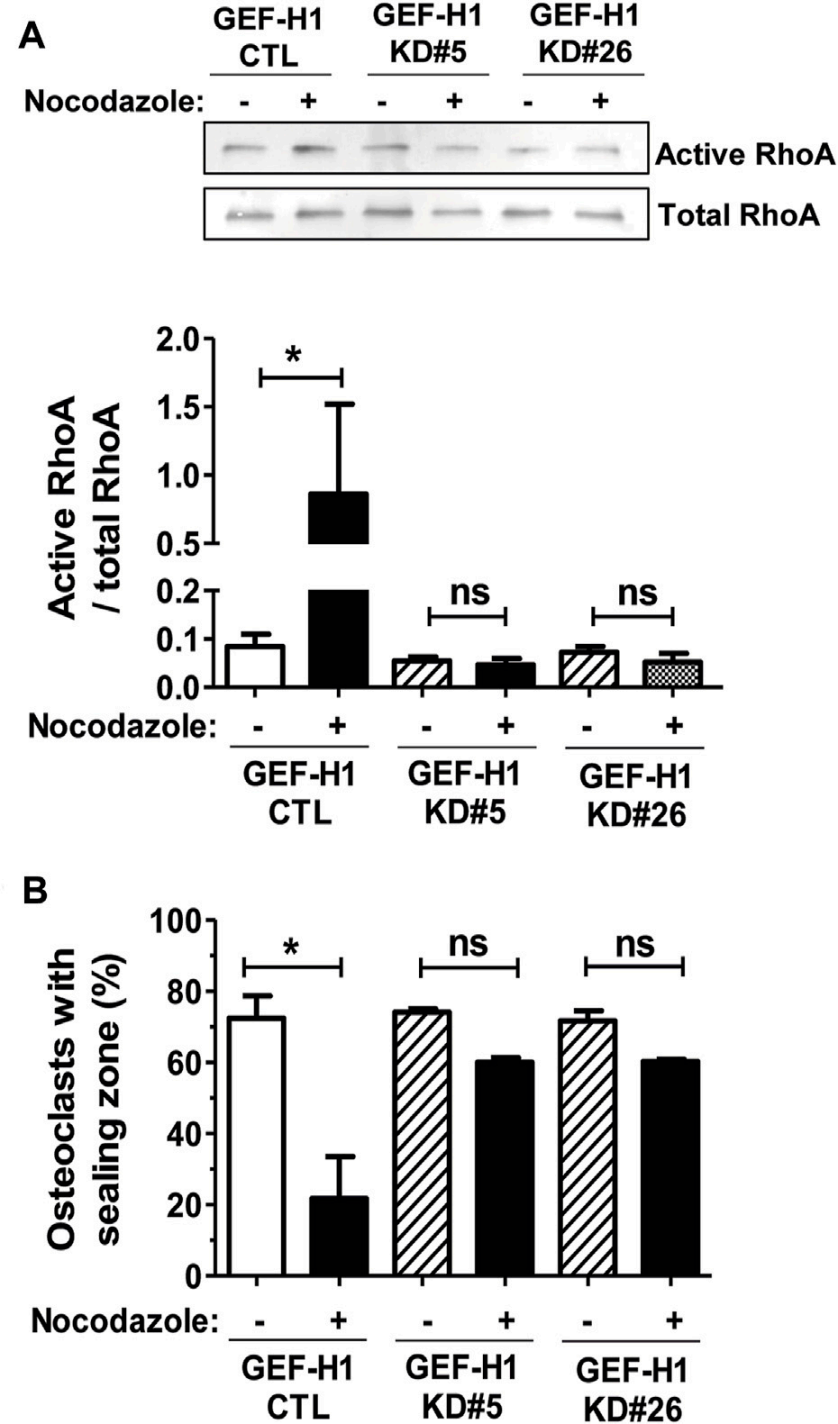
GEF-H1 is necessary for nocodazole-dependent RHOA activation and sealing zone loss on mineralized support. **(A)** Representative Western blot (top) and bar graph (bottom) showing mean ± s.e.m. active RHOA or **(B)** bar graph showing mean ± s.e.m. osteoclasts with sealing zone from control (CTL) and knock-down GEF-H1 osteoclasts derived from clones 5 (GEF-H1 KD#5) and 26 (GEF-H1 KD#26) treated (+) or not (−) with 10 µM nocodazole for 1 h (*n* = 3–5 independent experiments). ns, not significant; *, *p <* 0.05 as determined by one-tailed **(A)** or two-tailed **(B)** Mann-Whitney test.

To investigate the implication of GEF-H1 in the activation of RHOA upon osteoclast microtubule depolymerization, we compared the activation of the GTPase in response to nocodazole in CTL and GEF-H1 KD osteoclasts. Similar to BMM-derived osteoclasts, treatment of RAW264.7-derived CTL osteoclasts seeded on ACC with nocodazole led to a strong increase in the levels of active RHOA (Figure 4A). In contrast, the levels of active RHOA did not change in response to nocodazole in KD#5 and in KD#26 osteoclasts (Figure 4A). This shows that GEF-H1 plays a key role in the activation of RHOA upon osteoclast microtubule depolymerization. Since fine tuning of RHOA activity is critical for the organization of osteoclast actin cytoskeleton, we also examined the role of GEF-H1 in the formation and maintenance of sealing zones. Interestingly, in basal conditions, the proportion of osteoclasts presenting a sealing zone was comparable between CTL, KD#5 and KD#26 osteoclasts (Figure 4B). But in contrast with CTL osteoclasts, the sealing zones in KD#5 and KD#26 osteoclasts were not destabilized upon microtubule depolymerization by nocodazole (Figure 4B).

Overall, these data suggest that whereas GEF-H1 is required to the formation of osteoclasts, its sequestering on microtubules is necessary to ensure the optimal activity levels of RHOA and the maintenance of the sealing zone.

### GEF-H1 is necessary for osteoclast resorption activity

**FIGURE 5:**
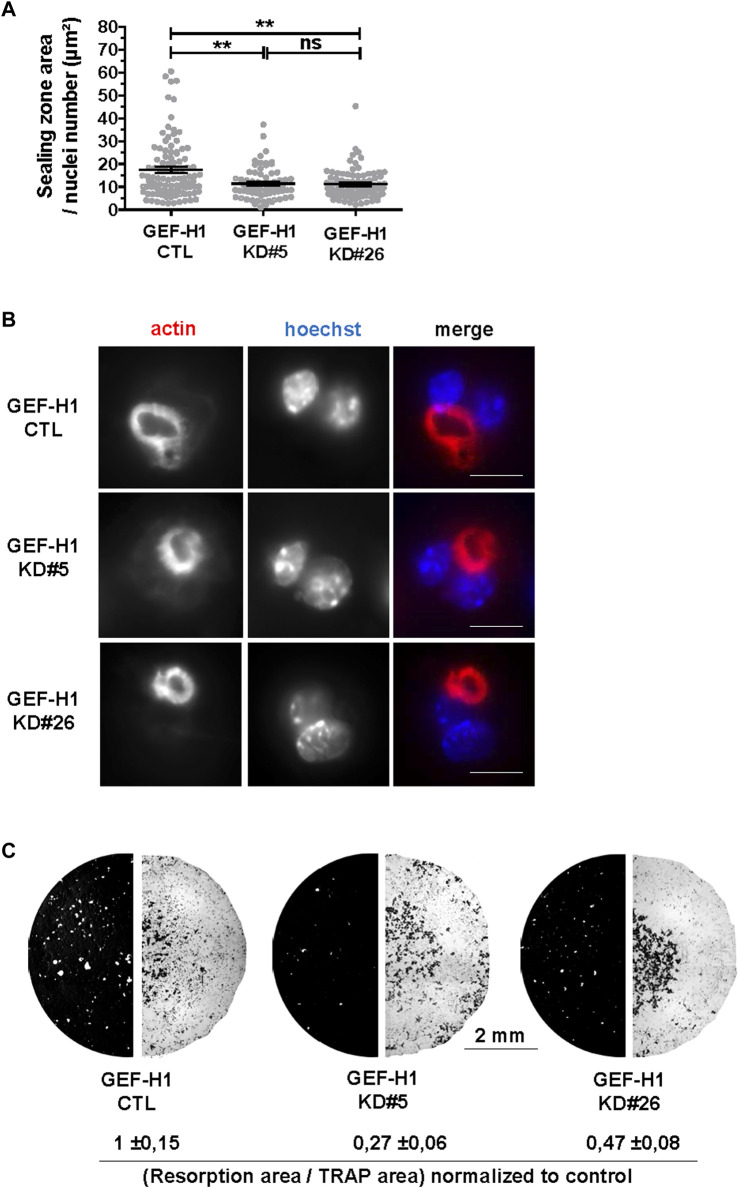
GEF-H1 is necessary for resorption of mineralized support. **(A)** Scatter graph showing mean normalized sealing zone area ±s.e.m. of control (CTL) or knock-down GEF-H1 osteoclasts derived from clones 5 (GEF-H1 KD#5) and 26 (GEF-H1 KD#26) (results from 4 different experiments). ns, not significant; **, *p <* 0.01 as determined by Kruskal-Wallis followed by Dunn’s multiple comparison test. **(B)** Confocal images of a single plane showing actin (red) and nuclei (Hoechst, blue) in control (CTL) or knock-down GEF-H1 osteoclasts derived from clones 5 (GEF-H1 KD#5) and 26 (GEF-H1 KD#26). Scale bars: 15 µm. **(C)** Representative resorption (left half circle, in white) and osteoclasts areas (right half circle, in black) visualized after Von Kossa and TRAP staining of Osteo Assay stripwell plated with control (CTL) or GEF-H1 knock-down osteoclasts derived from clones 5 (GEF-H1 KD#5) and 26 (GEF-H1 KD#26). Values underneath represent mean Von Kossa/Trap area signal ratio ±sem from 4 different wells.

The above data show that GEF-H1 contributes to osteoclast differentiation and to the regulation of their actin cytoskeleton. As osteoclast cytoskeleton is key for their bone resorption activity (Blangy et al., 2020), we further investigated how GEF-H1 influences this function. First, we observed that KD#5 and KD#26 osteoclasts on ACC exhibited smaller sealing zone size as compared to CTL osteoclasts (Figures 5A, B), despite the fact that the proportion of osteoclasts with a sealing zone was similar in CTL and KD osteoclasts (Figure 4B). Thus, notwithstanding it destabilizes the sealing zone when it is released from microtubules (Figure 4B), GEF-H1 is also required for the optimal assembly and/or stability of this structure. To confirm the functional relevance of this observation, we examined the effect of GEF-H1 on the resorption activity of osteoclasts. Consistent with the reduction in sealing zone size, we found that the resorption activity of GEF-H1 KD osteoclast was markedly reduced as compared to CTL (Figure 5C; Supplementary Figure S3).

These results suggest that GEF-H1 has to be finely tuned for optimal osteoclast activity.

## Discussion

The osteoclast microtubule network is well known to be essential for the formation and stability of the actin ring, and thus for osteoclast bone resorption activity. Former reports suggested that the activation of the GTPase RHOA was involved in the destabilization of the actin ring caused by microtubule disorganization; nevertheless, the mechanisms linking microtubules and RHOA activity in osteoclasts were unknown. Here we studied the function of GEF-H1 in osteoclast, as the only RHOA nucleotide exchange factor known to be regulated by microtubules. Our work shows that GEF-H1 is necessary for optimal osteoclast resorption function by regulating the organization of actin, but that the activity of GEF-H1 has to be tightly controlled by its association with microtubules to ensure optimal levels of RHOA activity and stability of the sealing zone.

To study the function of GEF-H1 in osteoclast, we used the CRISPR/Cas9 technology to generate RAW264.7 cells knock-out for *Arhgef2*, which encodes GEF-H1. RAW264.7 is a murine monocytic cell line commonly used as osteoclast precursors due to its ability to differentiate into osteoclasts in response to RANKL stimulation (Lampiasi et al., 2021). We observed that GEF-H1 knock-out cells were unable to go through differentiation, suggesting that GEF-H1 is necessary for this complex process. In brief, stimulation of monocytic precursors by RANKL induces their differentiation into pre-osteoclasts *via* downstream kinases which in turn activate transcription factors such as NF-KB, c-Fos and master regulator NFATc1. The latter regulates the expression of numerous osteoclast-specific molecules involved in the fusion of osteoclast precursors and the maturation of multinucleated osteoclasts. RHOA has been shown to be rapidly activated when RANKL binds to its receptor (Kim et al., 2021). Furthermore, loss- and gain-of-function of RHOA in the myeloid lineage of the mouse revealed that RHOA was required for ROCK-dependent activation of NF-KB and NFATc1, which is essential for osteoclast differentiation (Wang et al., 2023). Therefore, it is likely that reduced RHOA activation is responsible for the lack of differentiation of GEF-H1 KO RAW264.7 cells in osteoclasts, as the specificity of GEF-H1 for RHOA activation has been confirmed in numerous studies (Joo et al., 2021) since its discovery in the late 1990s (Whitehead et al., 1995; Ren et al., 1998). It would be interesting to further study which pathway is activated by GEF-H1 during early osteoclast differentiation induced by RANKL. In order to investigate further the role of GEF-H1 in the control of microtubule-dependent organization of osteoclasts actin cytoskeleton, we had to overcome the fact that GEF-H1 was essential for early osteoclast differentiation. We were able to restore osteoclast differentiation by mixing GEF-H1 knock-out cells with control cells, suggesting that low levels of the GEF-H1 are sufficient to go through the process.

**FIGURE 6:**
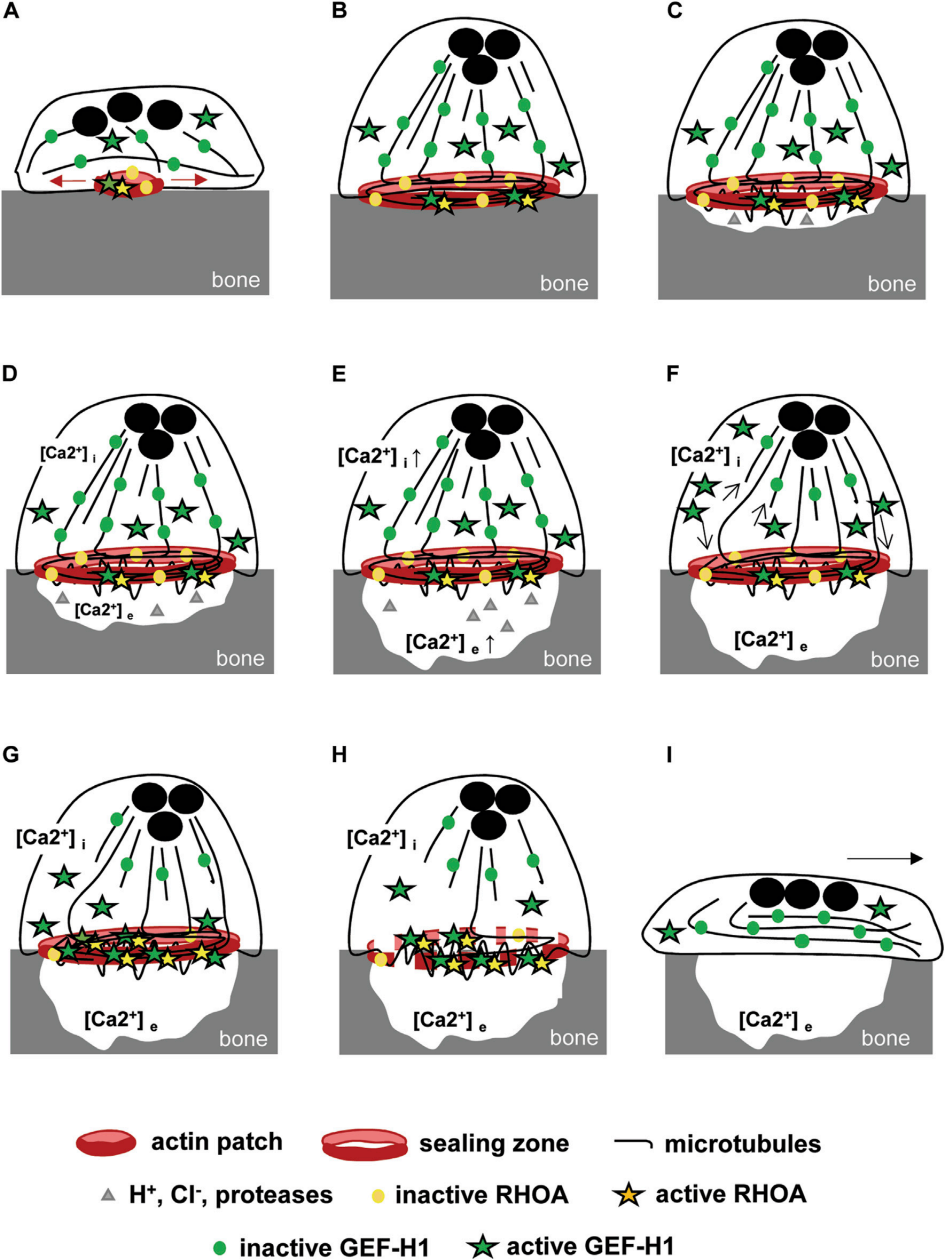
Model of GEF-H1-dependent bone resorption **(A)** Osteoclast adhesion to bone induces the formation of actin patches whose growth is dependent on RHOA activation by GEF-H1. **(B)** The resulting sealing zone is then stabilized by microtubules and **(C)** allows polarized osteoclasts to adhere to the bone and isolates a compartment in which protons, Cl^−^ and proteases are secreted. GEF-H1 is distributed between the microtubules and the cytosol, while RHOA is located mainly in the sealing zone. **(D)** Resorption is associated with the release of an increasing concentration of ionized calcium into the resorption lacuna. **(E)** It generates a parallel increase in intracellular calcium concentration by diffusion and *via* membrane receptors that **(F)** destabilizes locally microtubules (arrows) without altering the whole network and the sealing zone stability. As a result, GEF-H1 is released and **(G)** directed to the sealing zone where it is activated and **(H)** triggers RHOA and ROCK-mediated destabilization of the sealing zone. **(I)** Next, the osteoclasts spread out, which marks the start of the migration phase, enabling them to move to a new area of the bone surface to be resorbed.

The stability of the osteoclast actin ring relies on the microtubule network. We found that the depolymerization of microtubules disorganizes the podosome belt of osteoclasts plated on a non-mineralized substrate, confirming former results (Destaing et al., 2003). We also showed that the stability of the sealing zone also requires an intact microtubule network on a mineralized substrate, as mentioned as data not shown in a former study (Okumura et al., 2006). Moreover, the C3 toxin was found to prevent nocodazole-induced disorganization of the podosome belt, suggesting that RHOA activity plays a major role in this process (Destaing et al., 2005). Our results further show that the activation of the kinase ROCK is involved the destabilization of the podosome belt downstream of RHOA. As GEF-H1 is the only RHOA nucleotide exchange factor reported to be regulated by microtubules (Joo et al., 2021), it appeared as a highly relevant candidate regulator of the crosstalk between the actin cytoskeleton and microtubules in mature osteoclasts. In fact, GEF-H1 is unable to activate RHOA when trapped in an inactive state on polymerized microtubules; it was shown to be activated by a variety of mechanisms, both dependent (Chang et al., 2008; Lee et al., 2022) and independent (Kakiashvili et al., 2011; Meiri et al., 2014; Coló et al., 2023) of microtubule depolymerization in various cellular models. Through RHOA-ROCK-dependent actomyosin contraction and focal adhesion, GEF-H1 was shown to regulate diverse physio-pathological processes such as neutrophil shear-stress induced migration (Fine et al., 2016), endothelial permeability induced by mechanical stimulation (Birukova et al., 2010) or even neuron dendritic spines stability and size (Ryan et al., 2005). Although a substantial fraction of GEF-H1 was localized in the cytosol of osteoclasts, we showed that it is the release of microtubule-associated GEF-H1 that activates RHOA. As in other cell types, GEF-H1 activity is also regulated by its association with microtubules in osteoclasts. A biosensor developed to monitor the activity of GEF-H1 at the leading edge of migrating cells showed that local microtubule destabilization produced 5-μm-wide spatially constrained GEF-H1 activity. GEF-H1 is further regulated by Src phosphorylation to reach its highest catalytic state and its proper localization to the protrusion edge where RHOA is localised (Azoitei et al., 2019). In mammalian osteoclasts, a fraction of microtubules grow towards the top of the podosomes where they form a dense circular network over the podosome belt/sealing zone (Blangy et al., 2020). Following depolymerisation of these microtubules, GEF-H1 would be directly released at the podosome belt in which RHOA is enriched (Maurin et al., 2018) and may or may not need to be further regulated to activate RHOA. Whereas microtubules appeared essential to sequester GEF-H1 and modulate its activity to preserve the actin ring, the actual function of GEF-H1 in mature osteoclast cytoskeleton organization and function remained questionable. We found that sealing zones were more stable under nocodazole treatment in GEF-H1 knock-down osteoclasts as compared to controls. However, we observed that GEF-H1 knock-down osteoclasts exhibited a 40% decrease in normalized sealing zone size, as compared to control osteoclasts; this was expectedly associated with a reduction in the resorption activity of GEF-H1 knock-down osteoclasts. Thus, GEF-H1 does have an important function for the building of the sealing zone and for optimal osteoclast resorption activity. Bone degradation results from alternating phases of resorption by polarized osteoclasts with a sealing zone and phases of migration with a spread morphology without any specific actin structure (Saltel et al., 2004). How could our findings translate to *in vivo* osteoclasts? We propose that GEF-H1 could play a dual role during this process though a progressive release of its inhibited state on microtubules. First, during the resorption phase, moderate activation of RHOA by GEF-H1 would be required for the sealing zone to reach its maximal size; then, increasing activity of GEF-H1 could provoke the disorganization of the actin ring to allow osteoclast moving on from one resorption site to the next (Figure 6). In fact, actively resorbing osteoclasts dissolve the mineralized matrix of bone generating high extracellular ionized calcium concentration, to which they respond with a parallel rise in cytosolic calcium and a dramatic reduction of bone resorption (Zaidi et al., 1989). High calcium concentrations are known to induce microtubules depolymerization (Karr et al., 1980; Weisenberg et al., 1981; Miyauchi et al., 1990). Thus, osteoclast resorption activity could progressively lead to calcium-dependent depolymerization of microtubules and consequently release of more GEF-H1 to activate RHOA, resulting in the loss of the sealing zone. This could be achieved through ROCK-LIMK-dependent cofilin inactivation since it was shown that microtubules disruption deactivates cofilin which is enriched at the podosome belt *via* phosphorylation (Blangy et al., 2012; Zalli et al., 2016). Sealing zone destabilization marks the end of resorption at this site and the beginning of migration which will enable a new phase of resorption further down the bone surface. The microtubule network undergoes a reorganization and GEF-H1 is once again held inactive. In this model, GEF-H1 activity is tightly regulated in space and time by microtubule stability which varies oppositely with each phase. Recently, GEF-H1 cyclic release was also described for other biological processes finely regulated over time. It was shown that interphase microtubule disassembly activates GEF-H1 which promotes RHOA-dependent cell rounding at mitotic entry (Leguay et al., 2022). Similarly, cyclic GEF-H1 release induces a RHOA-mediated actomyosin contraction-dependent driving force that propels cells into confined spaces. Interestingly, oscillatory microtubules depolymerization was due to a persistent parallel increase of intracellular calcium concentration generated by mechanical confinement (Lee et al., 2022).

Overall, our data show that GEF-H1 is an important player in osteoclast resorption function through its regulation of RHOA activation. The fact that a simple decrease in its expression is sufficient to impair resorption suggests that this GEF would be an interesting target for controlling osteoclast activity, in particular in the context of osteolytic diseases in which osteoclasts are over-activated. Several GEFs of RhoGTPases have proved to be relevant therapeutic targets in various pathologies (Blangy, 2017), including osteolytic diseases (Vives et al., 2015; Mounier et al., 2020). In fact, targeting GEFs makes it possible to target more specific signaling pathways than inhibiting the GTPases themselves, the latter being involved in many key cellular functions. Interestingly, a peptide inhibiting RHOA activation by GEF-H1 has been shown to inhibit blood vessel leakage in a mouse model of uveitis (Mills et al., 2022), opening the door to a new therapeutic approach.

## Data Availability

The original contributions presented in the study are included in the article/Supplementary Material, further inquiries can be directed to the corresponding author.
