# Supplementary figures and images for: Bone resorption by osteoclasts involves fine tuning of RHOA activity by its microtubule-associated exchange factor GEF-H1
Source: Front Physiol. 2024 Jan 19;15:1342024. doi: 10.3389/fphys.2024.1342024 (PMC10834693; doi:10.3389/fphys.2024.1342024)

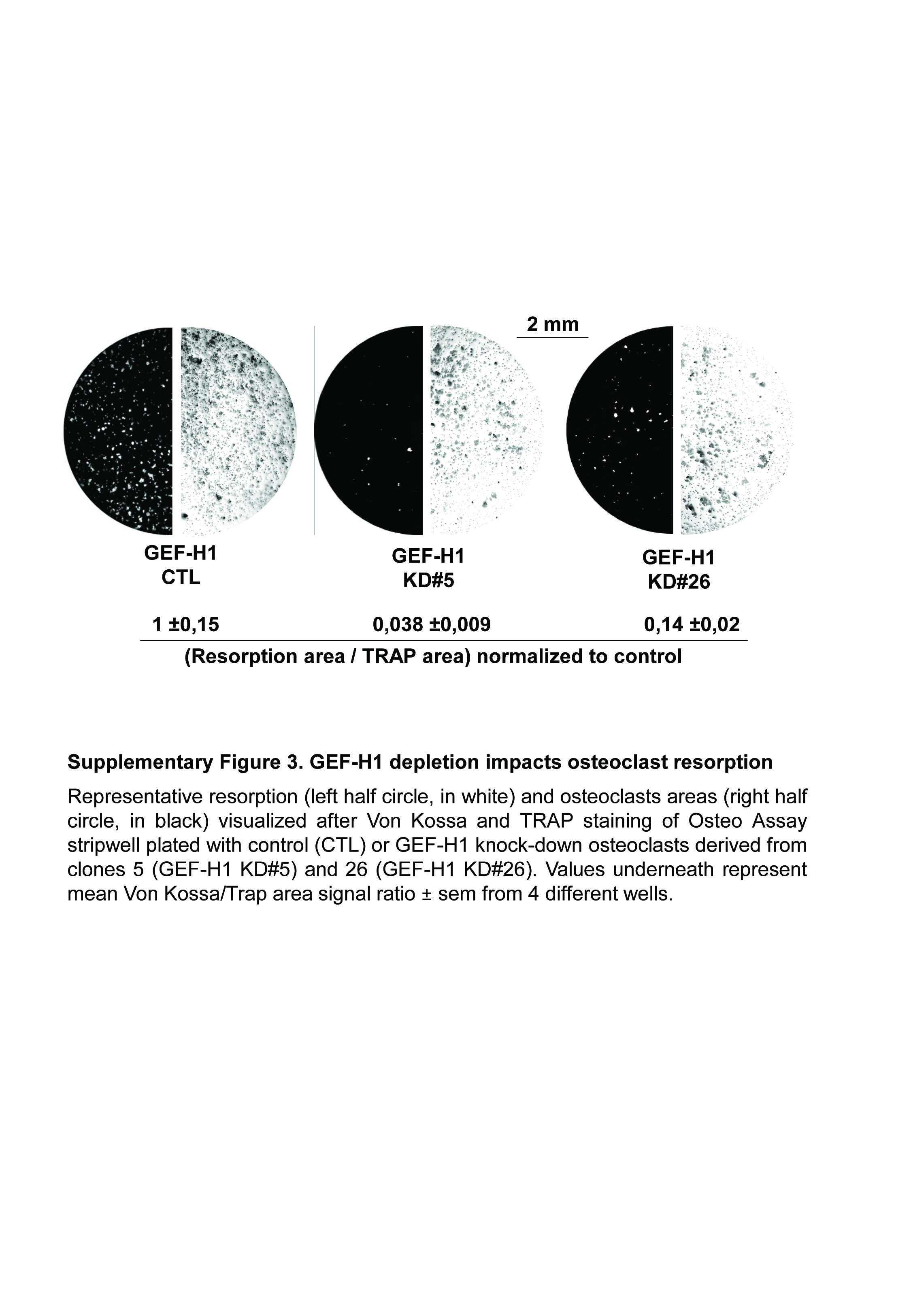

Supplement: Supplementary file 1 [file Image3.tif]

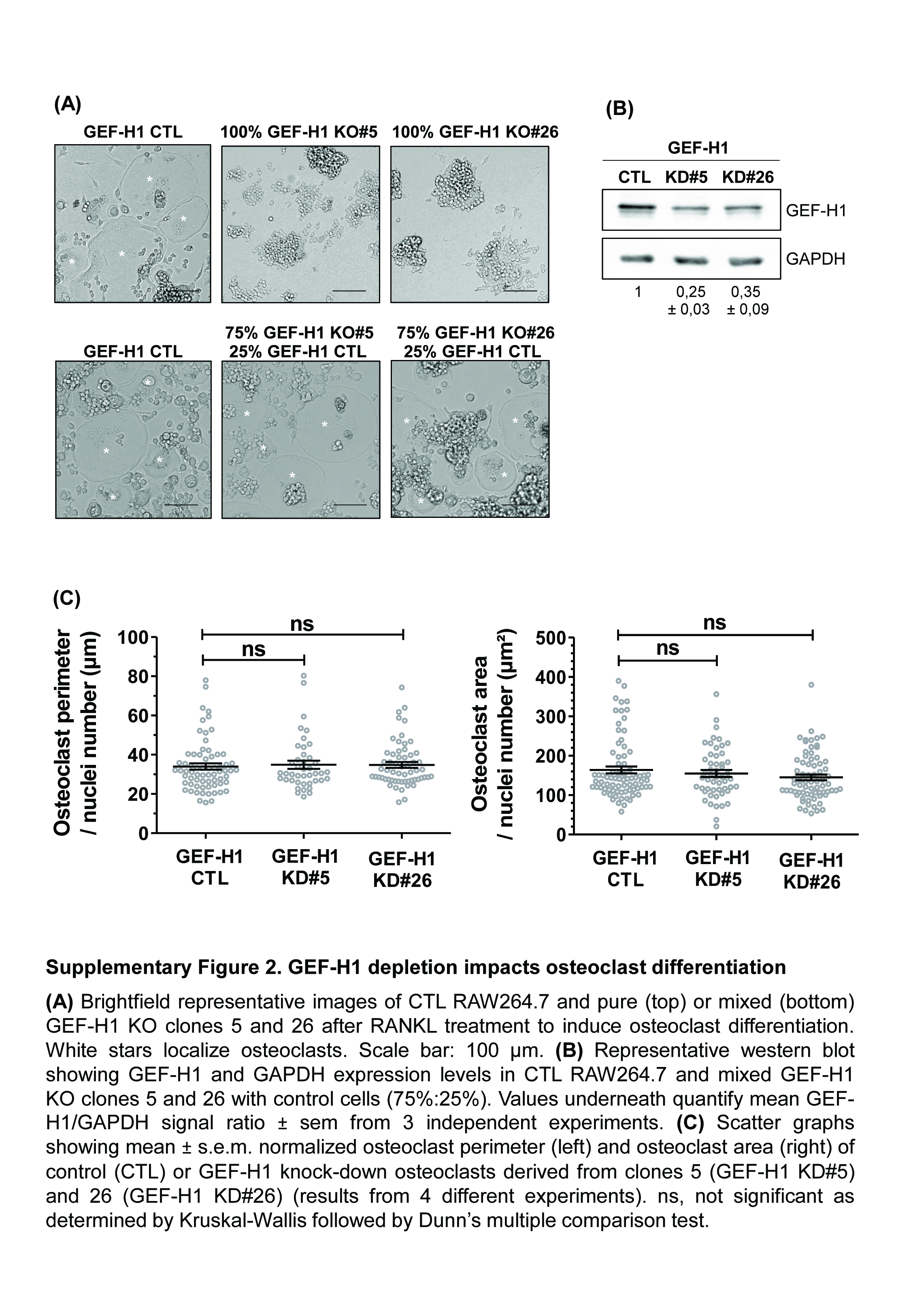

Supplement: Supplementary file 2 [file Image2.tif]

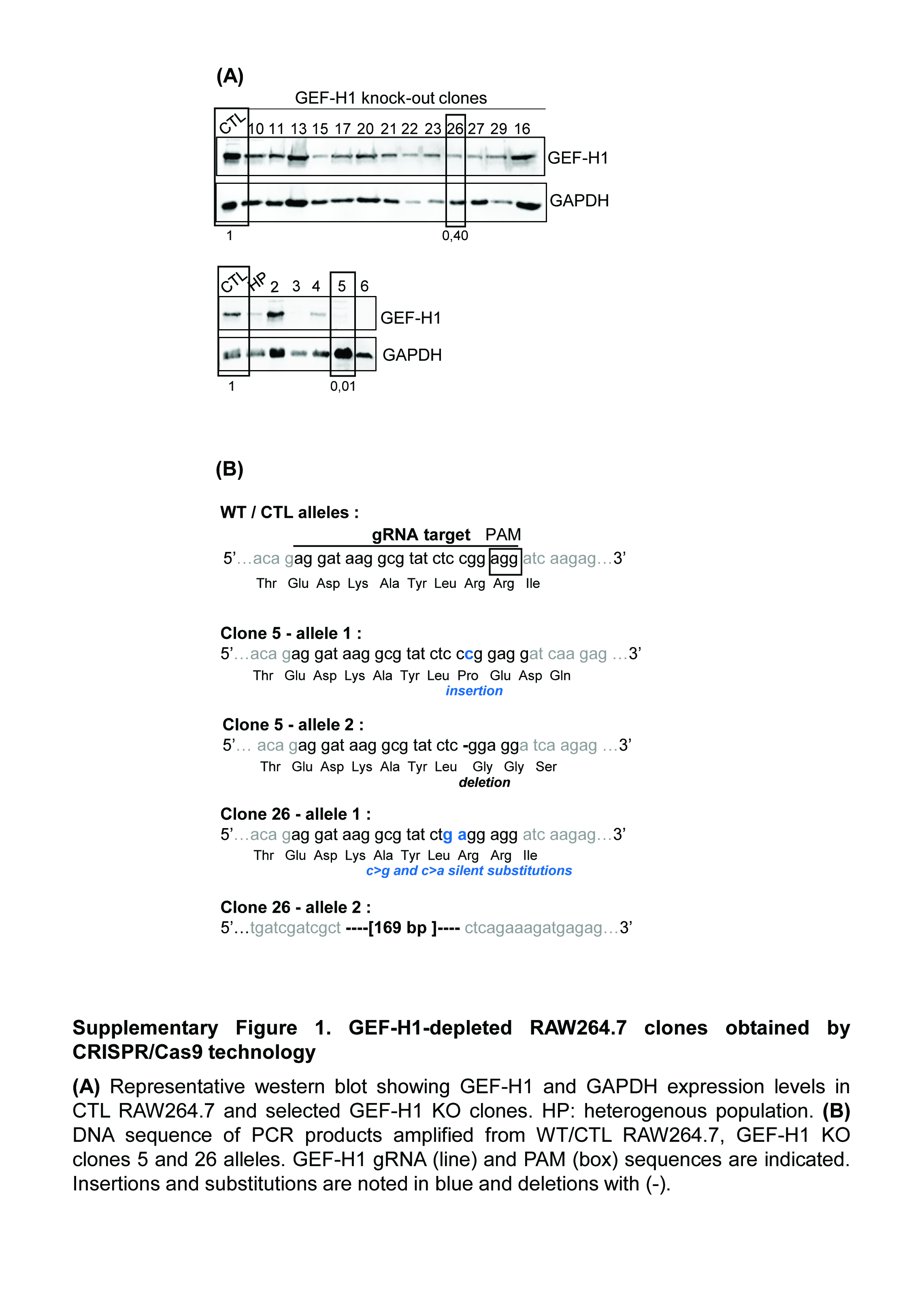

Supplement: Supplementary file 3 [file Image1.tif]
