# Supplementary material for: Bone resorption by osteoclasts involves fine tuning of RHOA activity by its microtubule-associated exchange factor GEF-H1
Source: Front Physiol. 2024 Jan 19;15:1342024. doi: 10.3389/fphys.2024.1342024 (PMC10834693; doi:10.3389/fphys.2024.1342024)
Supplement: Supplementary file 4 [file DataSheet1.pdf]

*Supplementary Material*

**Bone resorption by osteoclasts involves fine tuning of RHOA activity by its microtubule-associated exchange factor GEF-H1**

## SUPPLEMENTARY MATERIAL

### DNA sequencing analysis

Genomic DNA from Crispr/Cas9 Raw 264.7 GEF-H1 KO derived clones was extracted (Vazyme, #PD101) and the target site was amplified by PCR (New England Biolabs, #M0491S) with specific GEF-H1 forward CCGCAGAAGAGCAGAAGTCA and reverse AATCACACCTGAGCCTTC primers.
